# Effect of the enantiomeric structure of hydrophobic polymers on the encapsulation properties of a second near infrared (NIR-II) fluorescent dye for *in vivo* deep imaging†

**DOI:** 10.1039/d1ra08330a

**Published:** 2022-01-06

**Authors:** Kotoe Ichihashi, Masakazu Umezawa, Yuichi Ueya, Kyohei Okubo, Eiji Takamoto, Takashi Matsuda, Masao Kamimura, Kohei Soga

**Affiliations:** Department of Materials Science and Technology, Faculty of Advanced Engineering, Tokyo University of Science 6-3-1 Niijuku Katsushika Tokyo 125-8585 Japan masa-ume@rs.tus.ac.jp mail@ksoga.com; Tsukuba Research Laboratories, JSR Corporation 25 Miyukigaoka Tsukuba Ibaraki 305-0841 Japan

## Abstract

Over-thousand-nanometer (OTN) near-infrared (NIR) fluorophores are useful for biological deep imaging because of the reduced absorption and scattering of OTN-NIR light in biological tissues. IR-1061, an OTN-NIR fluorescent dye, has a hydrophobic and cationic backbone in its molecular structure, and a non-polar counter ion, BF_4_^−^. Because of its hydrophobicity, IR-1061 needs to be encapsulated in a hydrophobic microenvironment, such as a hydrophobic core of polymer micelles, shielded with a hydrophilic shell for bioimaging applications. Previous studies have shown that the affinity of dyes with hydrophobic core polymers is dependent on the polarity of the core polymer, and that this characteristic is important for designing dye-encapsulated micelles to be used in bioimaging. In this study, the dye–polymer affinity was investigated using hydrophobic polymer films with different chiral structures of poly(lactic acid). IR-1061 showed higher affinity for l- and d-lactic acid copolymers (*i.e.*, poly(dl-lactic acid) (PDLLA)) than to poly(l-lactic acid) (PLLA), as IR-1061 shows less dimerization in PDLLA than in PLLA. In contrast, the stability of IR-1061 in PDLLA was less than that in PLLA due to the influence of hydroxyl groups. Choosing hydrophobic core polymers for their robustness and dye affinity is an effective strategy to prepare OTN-NIR fluorescent probes for *in vivo* deep imaging.

## Abstract 2

The enantiomeric structure of PLA affects its affinity for OTN-NIR fluorescent IR-1061 dye and its robustness when forming hydrophobic core micelles.

## Graphical abstract

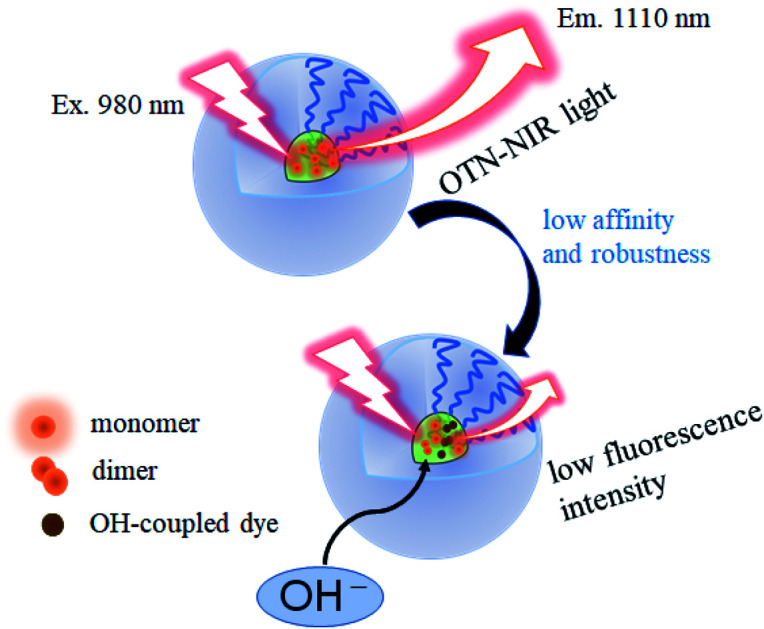

## 1. Introduction

Fluorescence bioimaging enables highly sensitive and dynamic observation of biomolecules and microstructures without ionizing radiation.^1^ Instead of fluorescence bioimaging in the ultraviolet (UV), visible, and shorter wavelength near-infrared (NIR) wavelength regions, the use of over-thousand-nanometer (OTN) NIR light is useful for the observation of deep tissues.^2^ The use of OTN-NIR light extends the observation depth in biological tissues from several millimeters for UV and visible wavelengths to 1–2 cm,^3^ because it is less scattered by the tissues.^2,4^ The wavelength range of 1000–1350 nm in the OTN-NIR has attracted much attention as the ‘second biological window’ of NIR.^5^

Currently, there is a wide variety of OTN-NIR fluorescent probes^6^ including quantum dots,^7–10^ single-walled carbon nanotubes,^11–20^ lanthanide-doped ceramic nanoparticles,^3,21–23^ and organic molecular dyes.^24–34^ Compared to inorganic nanoparticles, organic molecular dyes show great potential as probes with low toxicity and low bioaccumulation.^25^ IR-1061, a widely used OTN-NIR fluorescent dye, has hydrophobic and cationic (polarized) backbones in its molecular structure and a non-polar counter ion, BF_4_^−^, that can suppress vibration quenching.^35,36^ Replacing BF_4_^−^ with hydroxyl ions (OH^−^) results in IR-1061 being quenched due to the influence of the vibration modes of O–H bonds.^30^ Thus, probes using IR-1061 for fluorescence bioimaging applications should be designed for suppressing quenching.

One such probe, designed using IR-1061, consists of polymer micelles encapsulating this dye in their hydrophobic core.^24,28^ Previous studies have shown that the molecular state of IR-1061 varies between monomer and dimer depending on the polymer and solvent being used, as well as its concentration.^37^ In short, IR-1061 is a low polarity molecule that, while it can exist as monomers in environments with high affinity even at locally high concentration, tends to form dimers that show low fluorescence intensity in non-polar and hydrophobic environments.^37^ Previous studies have shown that the aggregate formation like dimers of organic dye molecules alters their optical properties such as emission intensity and wavelength.^37–39^ It is important to clarify the influence of the polymer on the dye's molecular state to better design highly emissive fluorescent probes using IR-1061 with less dimer formation.

Poly(lactic acid) (PLA), a well-known biocompatible and biodegradable polymer that is widely used to make fluorescent probes and drug delivery carriers in biomedical research,^40–44^ is well-suited for IR-1061 encapsulation based on its solubility parameter.^32^ Since lactic acid exists in two optical isomeric forms, l-lactate and d-lactate, there are different types of PLA: poly(dl-lactic acid), a d- and l-lactic acid copolymer (PDLLA); poly(l-lactic acid) (PLLA); and poly(d-lactic acid) (PDLA). While only PDLLA was used in our previous study,^32^ it has a different crystallinity from enantiomerically homogenous PLLA and PDLA^45^ and may show different imaging results of tumors as suggested when loaded with indocyanine green.^46^ The difference in the interaction *via* intramolecular polarity between PLA chains depending on their enantiomeric homogeneity has also been reported.^47^ We hypothesized that PLA's robustness and its affinity for IR-1061, which, in turn, affect the molecular state of IR-1061, depends on its chiral structure. In this study, we examined the difference in the molecular state of the dye depending on the enantiomeric structure of PLA when IR-1061-loaded micellar nanoparticles were synthesized using two types of poly(ethylene glycol)-*block*-(PEG-*b*-) PLA, PEG-*b*-PLLA and PEG-*b*-PDLLA.

## 2. Materials and methods

### 2.1 Materials

Acetonitrile (ACN) and dichloromethane (DCM) were purchased from Fujifilm Wako Pure Chemical Industries (Osaka, Japan). Epoxy resin was purchased from Nippon Chuko Boeki Co. (Osaka, Japan). IR-1061, PLLA (Mw: 5000), PDLLA (Mw: 5000) and bovine serum albumin were purchased from Sigma-Aldrich (MO, USA). Poly(ethylene glycol) methyl ether-*block*-poly(l-lactic acid) (PEG-*b*-PLLA, PEG Mw: 2000, PLLA Mw: 5000) and poly(ethylene glycol) methyl ether-*block*-poly(dl-lactic acid) (PEG-*b*-PDLLA, PEG Mw: 2000, PDLLA Mw: 5000) were purchased from NOF Co. (Tokyo, Japan). Dulbecco's phosphate-buffered saline (PBS) was purchased from Thermo Fisher Scientific Inc. (MA, USA). All the reagents were used without further purification.

### 2.2 Preparation of IR-1061-loaded polymer film

**Fig. 1:**
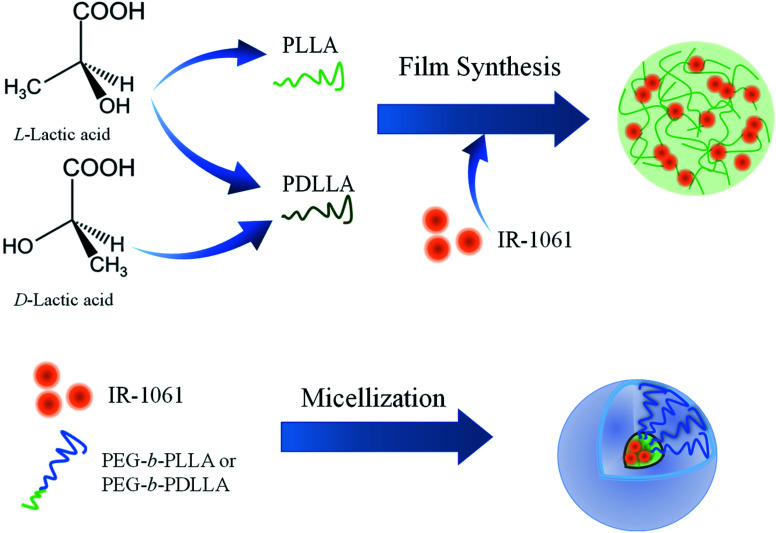
Schematic illustration of the design and preparation of OTN-NIR fluorescent dye-loaded polymer composite and micelles.The difference in the loading affinity of IR-1061 into PLA with and without enantiomeric homogeneity (PLLA and PDLLA) was investigated by analyzing the absorption spectra of the dye-loaded films.

Ten milligram of PLLA or PDLLA was dissolved in 0.9 mL of DCM and 1 μg of IR-1061 was dissolved in 0.1 mL of ACN. The solutions were then mixed. The mixed solution was dropped on a cube dish (bottom area: 1 cm^2^) under a nitrogen atmosphere using a glove box (UN-800F; UNICO, Ltd, Tsukuba, Japan). Dye-loaded PLLA or PDLLA films were prepared on dishes by evaporating the solvent overnight. After removing the organic solvents, the films were sealed with epoxy resin (Fig. 1).

### 2.3 Preparation of IR-1061-loaded polymer micelle

IR-1061-loaded PEG-*b*-PLA micelles were prepared using PEG-*b*-PLLA and PEG-*b*-PDLLA to compare the stability of the dye in the PLA cores with different chiral structure. PEG-*b*-PLLA or PEG-*b*-PDLLA (11.1 mg mL^−1^, 0.9 mL) and IR-1061 (0.1 mg mL^−1^, 0.1 mL) were mixed and dissolved in 1 mL of ACN. The mixture of PEG-*b*-PLA and IR-1061 was added dropwise into distilled water (4 mL) and then stirred overnight at 25 °C in a normal atmosphere to remove ACN and to obtain aqueous suspensions of micelles encapsulating IR-1061 (Fig. 1). The samples were purified by a centrifuge purification filter (MWCO 100 kDa, Merck Millipore, Darmstadt, Germany) at 20 000 g for 5 min, 4 times and dispersed in distilled water, PBS, and albumin solution (40 mg mL^−1^ on bovine serum albumin).

### 2.4 Characterization of IR-1061-loaded films and polymer micelles

The absorption spectra of the IR-1061-loaded films and polymer micelles were measured using an UV-visible-NIR spectrophotometer (V-770, JASCO, Japan). The obtained spectra were fitted with a Gaussian function using Igor Pro 8 software (Wavemetrics, Inc., OR, USA). A spectrometer (NIR-256-1.7, Avantes, Apeldoorn, Netherlands) was used to obtain the fluorescence spectra of IR-1061-loaded PEG-*b*-PLLA and PEG-*b*-PDLLA micelles from a 980 nm excitation source equipped with a fiber-coupled laser diode (SP-976-5-1015-7; Laser Components, Olching, Germany). The total concentration of IR-1061 loaded into the micelles was determined by the absorption (700–1300 nm). The loading efficiency was determined as *C*·*V*/*C*_0_·*V*_0_ × 100 (%), where *C*_0_ and *C* are the nominal and loaded concentrations of IR-1061, and *V*_0_ and *V* are the volumes before and after micellization (resuspension), respectively. The size of the IR-1061-loaded PEG-*b*-PLLA and PEG-*b*-PDLLA micelles was determined using dynamic light scattering (ELSZ-2000ZS, Otsuka Electronics Co., Osaka, Japan).

### 2.5 OTN-NIR fluorescence *in vivo* imaging of tumor model in mice

Colon-26 cells (1.0 × 10^5^ cells per mouse) were inoculated subcutaneously into the back of female 5 weeks-old BALB/c nu/nu mice (Japan SLC Inc., Shizuoka, Japan), fed the AIN-76A diet (Research Diets, NJ, USA). Seven days later, the mice were anesthetized under anesthesia and were treated with intravenous injection of an aqueous suspension (0.1 mL) of IR-1061-loaded PEG-*b*-PLA (50 mg mL^−1^) *via* the tail vein. OTN-NIR fluorescence images were collected using an *in vivo* OTN-NIR fluorescence imaging system SAI-1000 (Shimadzu Co., Kyoto, Japan). Animal care and experiments were conducted according to the guidelines on the care and use of laboratory animals at the Tokyo University of Science under approval of the Tokyo University of Science's Institutional Animal Care and Use Committee.

## 3. Results and discussion

### 3.1 Affinity of PLLA and PDLLA for IR-1061 in hydrophobic films

**Fig. 2:**
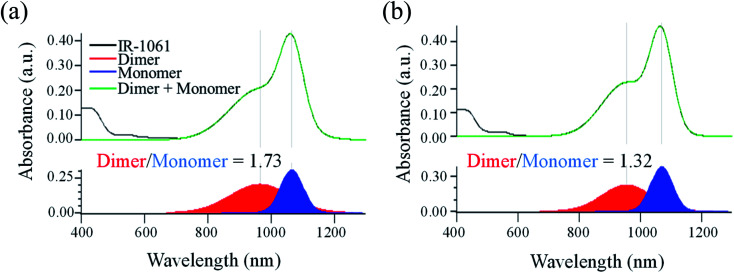
The ratio of IR-1061 dimers and monomers loaded in PLA films determined by its absorption spectra. Absorption spectra of IR-1061-loaded PLLA (a) and PDLLA (b) films. The peaks separated by Gaussian fitting for the absorption spectral data of each sample were also shown to determine the ratio of dimer and monomer of IR-1061 loaded in the films.

First, the influence of PLA's chiral structure on its affinity for IR-1061 was examined by determining the molecular states of the dye. The molecular state of the dye, however, is also affected by the surrounding molecules, such as water, in the medium. Thus, we first evaluated the effect of polymer properties on its affinity for IR-1061 by synthesizing and analyzing dye-loaded polymer films without hydrophilic molecules or aqueous media. The challenge with using films is the exposure of the dye to the air. When IR-1061 was exposed to air, BF_4_^−^ was replaced by OH^−^, resulting in the quenching of IR-1061 (Fig. S1†). This is due to the presence of water molecules in the atmosphere. Therefore, the dye-loaded polymer films were prepared under a nitrogen atmosphere. Dye-polymer affinity was evaluated by comparing the integrated absorbance of the dimer and the monomer separated using Gaussian fitting. IR-1061 dimers and monomers had peaks at approximately 950 and 1070 nm, respectively.^37^ The results showed that IR-1061 formed less dimers in PDLLA than in PLLA (Fig. 2), suggesting that IR-1061 showed higher affinity for PDLLA than PLLA. Although there is no difference in the solubility parameter, which is an indicator of the loading efficiency of dye molecules into polymeric materials,^32^ between PDLLA and PLLA based on their chemical structures, the enantiomeric homogeneity that contributes to the molecular arrangement of the polymer chains may affect the encapsulation properties of small molecules. Our results suggest that enantiomerically homogenized polymer chains can form a more stable assembly and can encapsulate only a small amount of other molecules in the chain.

### 3.2 *In vitro* stability of IR-1061-loaded polymer micelle with PLLA or PDLLA in its hydrophobic core

**Fig. 3:**
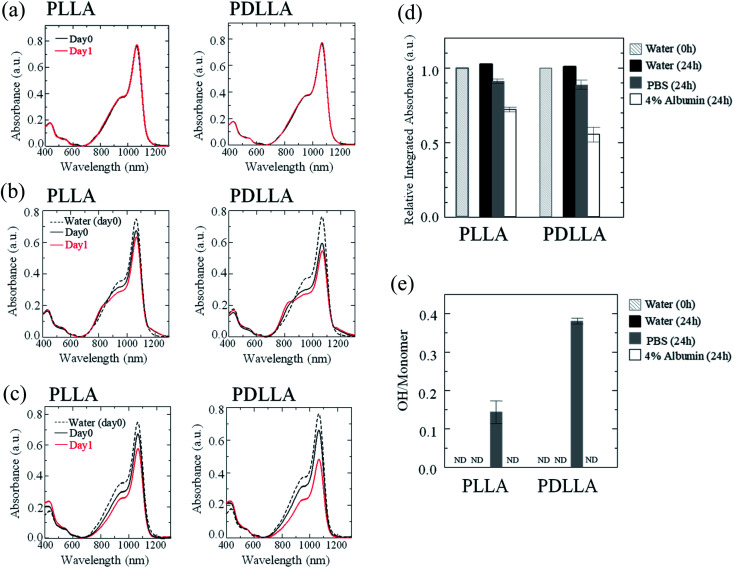
Stability of IR-1061 encapsulated in the polymer micelles of PEG-*b*-PLA dispersed in different media. Absorption spectra of polymer micelles encapsulating OTN-NIR fluorescent dye IR-1061 before (day 0) and after 24 h incubation in water (a), PBS (b), and 4% albumin solution (c). Total absorption of IR-1061 (600–1300 nm) in the IR-1061-encapsulated polymer micelles dispersed in indicated media (d) and the ratio of OH-coupled IR-1061 (ND: not detected) (e) are also shown. Error bars indicate standard deviation for each group (*n* = 3).

**Fig. 4:**
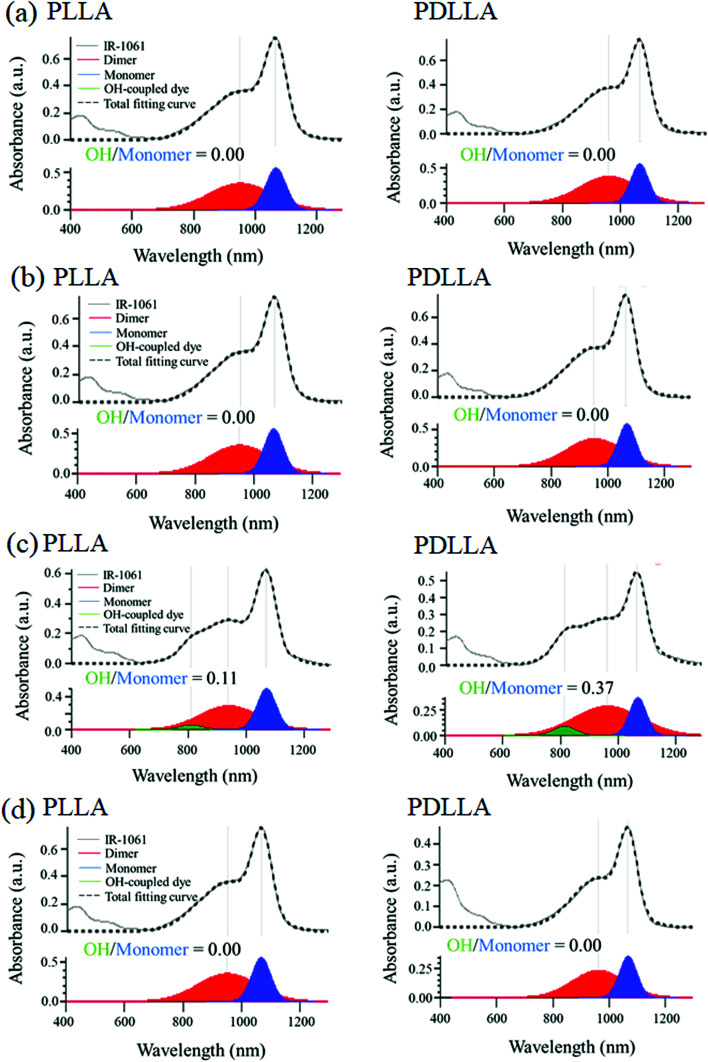
Deconvolution of absorption spectra of IR-1061-encapsulated PEG-*b*-PLLA and PEG-*b*-PDLLA micelles by Gaussian fitting. The peaks of dye monomers, dimers, and OH-coupled forms in the absorption spectra, separated by Gaussian fitting, of IR-1061-encapsulated PEG-*b*-PLLA (left) and PEG-*b*-PDLLA (right) micelles dispersed in different media. The data of (a) fresh samples in water, and the samples after incubation in (b) water, (c) PBS and (d) 4% albumin solution for 24 h are shown.

**Fig. 5:**
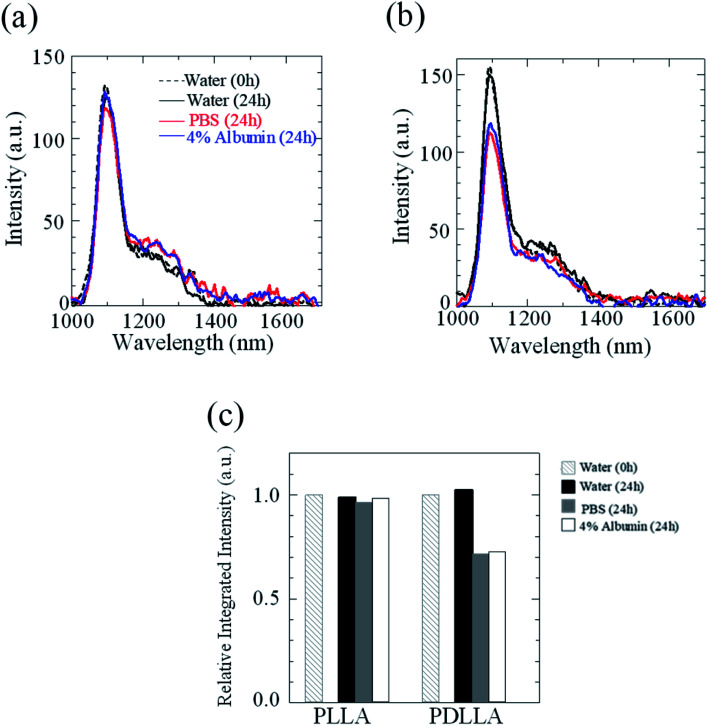
Effect of dispersion media on OTN-NIR fluorescence intensity of the IR-1061-loaded polymer micelles of PEG-*b*-PLA. Fluorescence spectra for IR-1061-loaded PEG-*b*-PLLA (a) and PEG-*b*-PDLLA (b) polymer micelles dispersed in indicated media for 24 h. Integrated intensities (1000–1400 nm) are also shown for IR-1061-loaded PEG-*b*-PLLA and PEG-*b*-PDLLA polymer micelles dispersed in indicated media for 24 h (c).

**Fig. 6:**
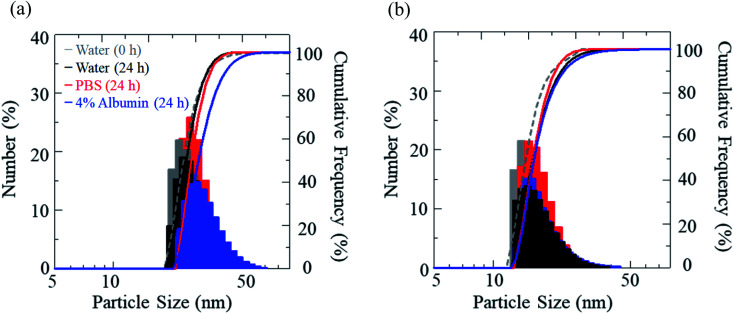
Size distribution of IR-1061-loaded PEG-*b*-PLLA micelles (a) and PEG-*b*-PDLLA micelles (b) before and after incubation in indicated media for 24 h.

The stability of IR-1061 in micelles depends not only on the affinity between the dye and the polymer, but also on the robustness of the dye with the polymer molecules that form the micelles. PLLA has higher crystallinity than PDLLA and can form a more robust hydrophobic core of micelles.^48^ PDLLA, which is amorphous and has a lower crystallinity compared to PLLA, is involved in the lower glass transition temperature, an indicator of polymer robustness, of PDLLA. We hypothesized that the difference in the robustness of PLLA and PDLLA in the hydrophobic core may affect the molecular state of the encapsulated dye. To test this hypothesis, IR-1061-loaded PEG-*b*-PLA micellar nanoparticles were prepared using PEG-*b*-PLLA and PEG-*b*-PDLLA to compare the stability of the dye in the PLLA and PDLLA cores, which have a PEG-shell, in micelles dispersed in aqueous media. PEG-*b*-PLLA and PEG-*b*-PDLLA co-polymers with the same chain lengths (molecular weights; PEG Mw: 2000, PLA Mw: 5000) were used to avoid the potential bias effects of the polymer molecular weights on the encapsulation properties of IR-1061 shown in previous investigations.^33^ The stability of IR-1061 in micelles was determined by comparing absorption (Fig. 3 and 4), OTN-NIR fluorescence (Fig. 5), and particle size (Fig. 6). At day 0, the loading efficiency of IR-1061 in the micellar nanoparticles, calculated from the data of concentration-dependent change in the optical absorbance,^30^ was 43% in both PEG-*b*-PLLA and PEG-*b*-PDLLA. The stability of the dye in the micelles dispersed in water, PBS, and an albumin solution was compared at 24 h after preparation. Albumin is a major protein constituent in the blood (approximately 40 mg mL^−1^ in human blood). After incubation in PBS or albumin solution at 25 °C for 24 h, the samples were analyzed without purification. As shown in Fig. 3d, the absorption of the dye in the PLLA and PDLLA cores dispersed in PBS and albumin solution decreased while the absorption of the dye in the PLA core remained unchanged when dispersed in water. In the albumin solution, the absorption of IR-1061 monomers and dimers in the PDLLA core was lower than that of its absorbance in the PLLA core. Previous studies have shown that albumin can disrupt micellar structures and affect their retention of encapsulated small molecules.^32,49,50^ Our results also suggest that albumin interacts with PEG-*b*-PDLLA, which has low robustness and reduces the stability of the micelles, suggesting that albumin enhanced the leakage of IR-1061 from the polymer core (Fig. 3d). Notably, a small shoulder appeared at about 800 nm, which indicates the coupling of hydroxyl ions with IR-1061,^31,32^ in the dye-encapsulated PEG-*b*-PLA micelles when incubated in PBS for 24 h (Fig. 3e). The ratio of OH-coupled IR-1061, which is a quenching factor of the dye, was higher in PEG-*b*-PDLLA than in PEG-*b*-PLLA (Fig. 3e and 4). This may be due to the larger influx of water into the PDLLA core because of its lower robustness. The luminescence intensity of the dye in PDLLA dispersed in PBS or albumin solution was lower than that of the dye in PLLA. These decreases in fluorescence intensity are consistent with the decrease in the absorption of the monomer of the dye (Fig. 5). The particle size of the dye-encapsulated PEG-*b*-PLLA micelles and PEG-*b*-PDLLA micelles was 26.5 ± 0.5 nm and 27.7 ± 0.3 nm in water at day 0, respectively. After incubation for 24 h, the sizes of the dye-encapsulated PEG-*b*-PLLA micelles were 26.6 ± 0.5 nm, 30.1 ± 1.3 nm, and 29.7 ± 1.3 nm in water, PBS, and 4% albumin solution, while those of PEG-*b*-PDLLA were 28.3 ± 0.4 nm, 27.6 ± 1.3 nm, and 27.9 ± 2.1 nm in water, PBS, and 4% albumin solution, respectively. These results suggested that, even after incubation in PBS and albumin solution for 24 h, the sizes of the dye-encapsulated PEG-*b*-PDLLA and PEG-*b*-PLLA micelles were stable at approximately 25–30 nm (Fig. 6).

### 3.3 *In vivo* OTN-NIR fluorescence imaging in mice

**Fig. 7:**
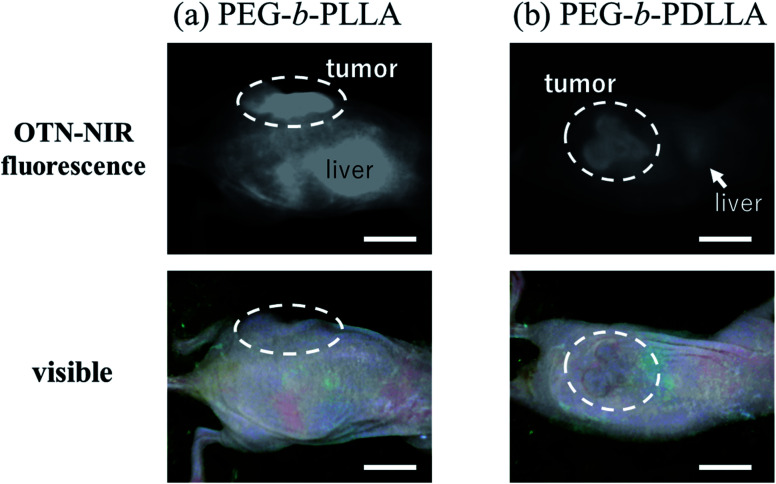
OTN-NIR fluorescence *in vivo* imaging of live mice with tumor models. OTN-NIR fluorescent IR-1061-encapsulated (a) PEG-*b*-PLLA and (b) PEG-*b*-PDLLA micelles (5 mg micelles containing 2.4 μg of IR-1061) dispersed in PBS (0.1 mL) were injected. The visible and OTN-NIR fluorescence images, collected under 980 nm light irradiation (0.2 W cm^−2^) with an integration time of 500 ms, of mice at 24 h after the intravenous injection are shown. Scale bars indicate 10 mm.

Images of demonstrative experiments in mice suggested that PEG-*b*-PLLA micelles encapsulating IR-1061 showed higher retention of fluorescence in *in vivo* tissues, including tumor model lesions, after intravenous injection compared to PEG-*b*-PDLLA micelles encapsulating the dye (Fig. 7). The higher retention of the dye-loaded PEG-*b*-PLLA was possibly owing to its higher stability. Therefore, dye molecules encapsulated in polymeric micelles with an enantiomerically homogenous PLLA hydrophobic core is expected to be a stable and biocompatible fluorescent contrast agent for visualizing targets in deep areas of biological tissues. The intensity per tumor area was 1.6-fold higher in the result of PEG-*b*-PLLA compared to that of PEG-*b*-PDLLA, while the tumor/liver ratio of the intensity was slightly (1.1-fold) higher in the case of PEG-*b*-PDLLA compared to that of PEG-*b*-PLLA. Further investigation of probe design including ligand modification is needed to increase the specificity for tumor lesions.

## 4. Conclusion

In the present study, we evaluated the differences in the affinity and robustness of IR-1061, an OTN-NIR fluorescent dye, in PLA, a biocompatible polymer, with different optical isomers, namely PDLLA and PLLA. The dye formed less dimers in PDLLA than in PLLA, suggesting that PDLLA has a higher affinity for IR-1061 than PLLA. In contrast, IR-1061 was less stable when encapsulated in the hydrophobic PDLLA core of micellar nanoparticles, which have a PEG-shell, than in the PLLA core. IR-1061 formed a more OH-coupled form, which is a quenching factor, in the PDLLA core than in the PLLA core of micelles. These results show the need to select hydrophobic chains with high robustness as well as high affinity to the dye, which are dependent on the enantiomeric structure of the hydrophobic core polymer, to prepare probes that show high fluorescence intensity.

## Author contributions

KS was the main project leader and conceived the overall research idea. KI, MU, and YU performed the experiments and data collection. KO, ET, MT, and MK were substantially involved in data analysis. KI and MU drafted manuscript. All authors read and approved the final manuscript prior to submission.

## Conflicts of interest

The authors have no conflict of interests to declare related to this manuscript.

## Supplementary Material

RA-012-D1RA08330A-s001
