# Supplementary material for: Effect of the enantiomeric structure of hydrophobic polymers on the encapsulation properties of a second near infrared (NIR-II) fluorescent dye for in vivo deep imaging
Source: RSC Adv. 2022 Jan 6;12(3):1310–8. doi: 10.1039/d1ra08330a (PMC8979197; doi:10.1039/d1ra08330a)
Supplement: RA-012-D1RA08330A-s001 [file RA-012-D1RA08330A-s001.pdf]

Electronic Supplementary Material (ESI) for RSC Advances.
This journal is © The Royal Society of Chemistry 2021

Supporting Information

Effect of Enantiomeric Structure of Hydrophobic Polymer on Encapsulation Property of the Second Near Infrared (NIR-II) Fluorescent Dye for *In Vivo* Deep Imaging

Kotoe Ichihashi,[1] Masakazu Umezawa,[1,*] Yuichi Ueya,[2] Kyohei Okubo,[1] Eiji Takamoto[2], Takashi Matsuda,[2] Masao Kamimura,[1] and Kohei Soga[1,*]

[1] Department of Materials Science and Technology, Faculty of Advanced Engineering, Tokyo University of Science, 6-3-1 Niijuku, Katsushika, Tokyo 125-8585, Japan; [2] Tsukuba Research Laboratories, JSR Corporation, 25 Miyukigaoka, Tsukuba, Ibaraki 305-0841, Japan.

*Corresponding authors: masa-ume@rs.tus.ac.jp (M. U.) and mail@ksoga.com (K. S.)

1. Characterization of IR-1061-Loaded Polymer Film and in Acetonitrile (ACN) Prepared in Air Containing Water

Poly(lactide-*co*-glycolic acid) (PLGA, Mw: 7,000−17,000, 5 mg; Sigma-Aldrich, MO, USA) and IR-1061 (0.05 mg) were dissolved in 0.5 mL of ACN and placed on a glass slide (S1112, Matsunami Glass Ind., Ltd., Japan) in the air. The films were prepared on the glass slide by removing ACN by evaporation for 30 min. The absorption spectra of IR-1061-loaded PLGA films were measured using an ultraviolet-visible-NIR (UV-VIS-NIR) spectrophotometer V-770 (JASCO, Japan).

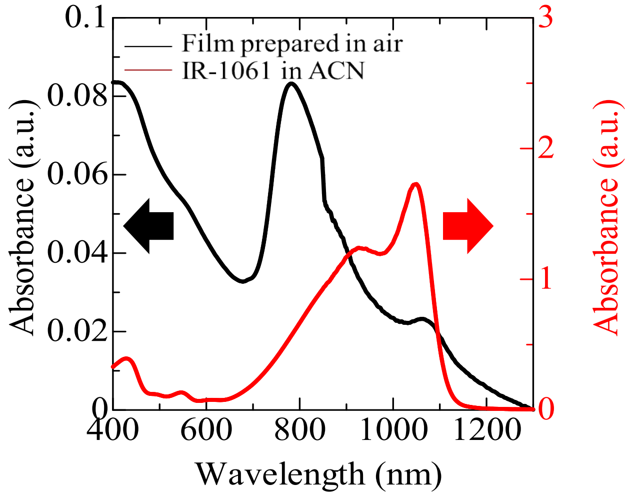

Supplementary Fig. S1. Absorption spectra of IR-1061 dissolved in ACN (red) and loaded in a polymer (PLGA) film prepared in air (black). The absorption spectrum of the dye-loaded polymer film in the air was peaked at 780 nm, suggesting that IR-1061 was majorly coupled with hydroxyl ions derived from water (Y. Ueya et al., *RSC Adv.*, 2021, **11**, 18930 [31]; *Anal. Sci.*, 2021, in press [33]). Therefore, the dye-loaded polymer films were prepared under nitrogen atmosphere in the glove box in following experiments in this study.
